# A Nitrogen-Rich Triazine-Based Covalent Organic Polymer Decorated with Nickel for Supercapacitors

**DOI:** 10.1021/acsomega.5c13542

**Published:** 2026-07-09

**Authors:** Aditya Bhat, Nakul Desai, Venkatachalam Hillemane, Sudhakar Yethadka Narahari

**Affiliations:** Manipal Institute of Technology, 76793 Manipal Academy of Higher Education, Manipal 576104, India

## Abstract

A nitrogen-rich triazine-based covalent organic polymer (COP) was made from melamine and cyanuric chloride through a solid-state grinding process. The poor electrochemical performance due to low nitrogen content and structural instability has been overcome in this process. To enhance active sites, nickel was doped into the COP. Fourier transform infrared (FTIR) analysis confirmed successful polymerization, the release of chlorine, and the presence of nickel oxide. X-ray diffraction (XRD) patterns indicated that the crystalline form of the COP remained intact after nickel incorporation. Scanning electron microscopy (SEM) images displayed a change from smooth to a rough, granular texture due to nickel’s interaction within the polymer matrix. Electrochemical tests demonstrated the better performance of the nickel-doped COP, showing a specific capacitance of 40 Fg^–1^ at 5 mVs^–1^ (cyclic voltammetry, CV) and 23 Fg^–1^ at 0.1 Ag^–1^ (galvanostatic charge/discharge, GCD), with a maximum power density of 835 Wkg^–1^ at 5 mVs^–1^. Notably, the electrode showed great cycling stability, maintaining almost 70% of its initial capacitance after repeated charge and discharge cycles. The results show that triazine-based COPs doped with nickel have potential for application in the use of supercapacitors due to their stability, efficiency, and duration.

## Graphical abstract

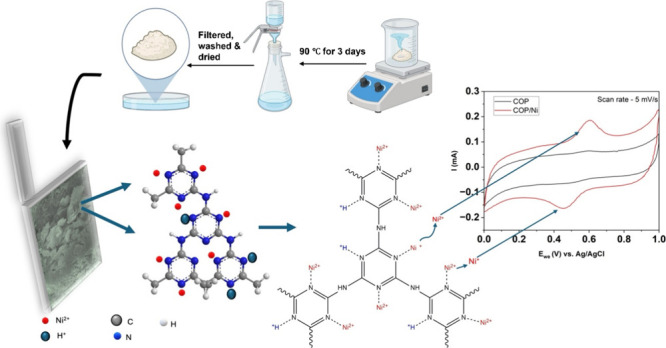

## Introduction

As the world is transforming toward more sustainable energy sources, the demand for clean and renewable energy has increased significantly. Supercapacitors hold a great deal of potential as storage units for energy that bridge the gap between batteries and traditional capacitors. Supercapacitors are being incorporated into electric vehicles, pulse power systems, and other technical systems because of their high power density, quick charge–discharge cycles, and long cycling stability. They have not been implemented commonly because of their low energy density, self-discharge, and low output at times. Covalent organic polymers (COPs) are novel materials that may be implemented as electrode materials to improve upon these problems. COPs are a flexible paradigm for improving the storage for electrochemical energy because of their lightweight design, large surface area, and strong covalent frameworks necessary to store ions.^1,2^

The primary determining feature for boosting a COP’s efficiency in supercapacitors is its stability and porosity within the electrochemical reaction. The pore structure is determined by the building blocks and their structures. The use of rigid components in the forms of imines, triazines, and boroxines allows for the preparation of long, porous frameworks. Formation of an ideal mesoporous structure (pore size >2 nm) greatly increases supercapacitor performance by improving ion diffusion and providing a high surface area that leads to an increase in the specific capacitance and faster charge–discharge cycles.^3,4^

The nitrogen content in a COP affects various properties that include porosity. Moderate nitrogen content in COPs enhances mesopore formation, which consequently enhances the electrochemical performance. Nitrogen sites also enable effective metal ion incorporation by providing strong binding sites, leading to enhanced redox activity and pseudocapacitance. However, excess nitrogen content can block the micropores, which affects the performance of supercapacitors.^5,6^

Despite their notable advances in the development of COPs for supercapacitor applications, certain frameworks such as c-PAF, Hex-Aza-COF-3, and CNT/NKCOF-2 have demonstrated promising electrochemical performance, yet their potential remains unexplored in the context of synergistically combining high nitrogen content and tailored mesoporosity.^7–10^

The choice of triazine-based COPs synthesized from melamine and cyanuric chloride as the monomers was due to their nitrogen-rich framework as well as planar and stable structure. They have high chemical and thermal stability, provide active sites for metal ion incorporation, promote mesoporosity for ion transport, and improve conductivity through π-conjugation, making them ideal for supercapacitor applications.^11,12^

In addition to the above requirements, metal ion incorporation in supercapacitors significantly enhances their performance by introducing additional redox-active sites, which increase the pseudocapacitance behavior. Incorporation of metal ions increases electrical conductivity, enables faster charge transfer, and reduces resistance, boosting specific capacitance. Further, metal ions create lattice defects or distortions that improve surface area and ion-diffusion rates. All these changes synergistically increase energy density, power density, and cycling stability of supercapacitors.^13,14^ a In the present work, we have chosen nickel as a dopant for the COP for its high redox activity, pseudocapacitance behavior, and mainly multiple oxidation states that enable high-efficiency charge storage by a reversible redox reaction^15,16^ . The novel strategy of a COP doped with nickel addresses the essential balance between the nitrogen-rich framework and the mesoporous structure for a high-performance supercapacitor. Optimizing nitrogen loading enables the material to accommodate steady dopant incorporation of nickel ions that creates redox-active sites that boost conductivity, pseudocapacitance, and stability. The research fills a fundamental research gap by integrating porosity and metal ion electrochemical activity for a robust metal-COP-based electrode material for energy storage.

## Materials and Methods

### Materials

Cyanuric chloride (98.5%), melamine, and acetone were purchased from Loba Chemicals. Dimethyl sulfoxide (DMSO) and ethanol were purchased from Merck. Nickel­(II) nitrate hexahydrate (99%) was purchased from Sigma-Aldrich. Polyvinylidene fluoride (PVDF, 99%) was procured from Merck, India. *N*-Methyl-2-pyrrolidone (NMP, 99%) was purchased from Loba Chemicals.

### Synthesis of Triazine-Based COP

The triazine-based COP was synthesized through a solid-state grinding method. Equimolar amounts of melamine and cyanuric chloride were thoroughly ground using a mortar and pestle until a fine homogeneous powder was obtained. The resulting mixture was stirred continuously at 60–70 °C for 24–48 h, yielding a pale-yellow solid. This crude product was washed successively with DMSO and ethanol, followed by filtration. To remove unreacted melamine, the filtered powder was stirred in hot water for 1 h, filtered again, and dried in a hot air oven at 90 °C for 4 h.

### Synthesis of Ni-COP

For nickel incorporation, an equal weight ratio of the prepared COP and nickel nitrate hexahydrate was ground thoroughly to ensure uniform mixing. The mixture was then stirred at 60–70 °C for 3 h. After completion of the reaction, the product was washed with acetone to remove excess metal salt and dried to obtain the final nickel-doped COP (Ni-COP).

### Three-Electrode Setup

For electrochemical studies, the prepared material was coated on the surface of stainless steel (1 × 1 cm), by preparing a slurry consisting of the material/composite, carbon, and PVDF in an 8:1:1 ratio, while keeping NMP as the solvent. The electrodes were dried and used for the electrochemical studies using a three-electrode setup, wherein Ag/AgCl was used as the reference electrode and a platinum electrode was used as the counter electrode, and they were all studied using a Biologic SP-50e electrochemical workstation.

The specific capacitance of the electrode material was calculated from the CV curves using the following equation:

$$C_{s}=\frac{\int I(V)dV}{2\mathrm{mv}\Delta V}$$

where *C*_s_ is the specific capacitance, ∫*I*(*V*)­d*V* is the integrated area of CV curve, *m* is the mass of the active material, *v* is the scan rate (V/s), and Δ*V* is the potential window (V).

Additionally, for the calculation of Cs from the GCD curves, the following equation was used:

$$C_{s}=\frac{I\times \Delta t}{m\times \Delta V}$$

where *I* is the discharge current (A), Δ*t* is the discharge time (s), *m* is the mass of the active material, and Δ*V* is the potential window (V).

The energy density and power density were calculated using the following equations:

$$E_{d}=\frac{C_{s}(\Delta V{)}^{2}}{2}$$

$$P_{d}=\frac{E_{d}}{\Delta t}$$

where *C*_s_ is the specific capacitance (F/g), Δ*V* is the potential window (V), and Δ*t* is the discharge time (s).

### Supercapacitor Fabrication

The performance metrics from the three-electrode system are investigated thoroughly, and the appropriate electrolyte and material composition are selected for device fabrication.^18^ The supercapacitor fabrication was done by coating the electrode surfaces of the Swagelok cell with the composite material on both sides as a symmetric configuration. Whatman filter paper was then soaked in 0.1 M H_2_SO_4_ electrolyte and placed between the two electrodes, and the Swagelok cell was assembled and connected to the Biologic SP-50e.

### Characterization Techniques

Scanning electron microscopy (SEM) analysis of ZESIS (EVO MA18) was performed to study the surface morphology. XRD was performed via an X-ray diffractometer (Rigaku Mini flex 600 (fifth generation)) with a scan rate of approximately 0.75 per second. FTIR spectra were obtained from an FTIR spectrophotometer (Shimadzu 400 MHz Bruker spectrometer) in the range of 400–4000 cm^–1^. The surface area was determined via Brunauer–Emmett–Teller (BET) analysis (BELSORP mini-X, BELPREP VAC 111, Japan). X-ray photoelectron spectroscopy (XPS) was performed on a Thermo Fisher instrument (Nexsa base, K-alpha), model no. 9419224062. To evaluate the performance of the electrodes for supercapacitor studies, cyclic voltammetry (CV), electrochemical impedance spectroscopy (EIS), and galvanostatic charge–discharge (GCD) were performed using a Biologic SP-50e in 0.1 M H_2_SO_4_.

## Results and Discussion

### FTIR Studies

The FTIR spectra of melamine, cyanuric chloride, COP, and Ni-COP depicted in Figure 1 confirm the successful formation of the polymer and subsequent metal coordination. Melamine shows N–H stretching around 3400–3300 cm^–1^ and triazine ring vibrations around 1560 and 1460 cm^–1^, while cyanuric chloride exhibits C–Cl stretches around 850–700 cm^–1^. In the COP, the disappearance of C–Cl (850 cm^–1^) bands and the emergence of C=N around 1650 cm^–1^ and C–N around 1300–1000 cm^–1^ bands confirm the formation of the triazine-based polymer. Ni-COP retains these features, with additional peaks around 600–400 cm^–1^, indicating Ni–N coordination.

**Figure 1:**
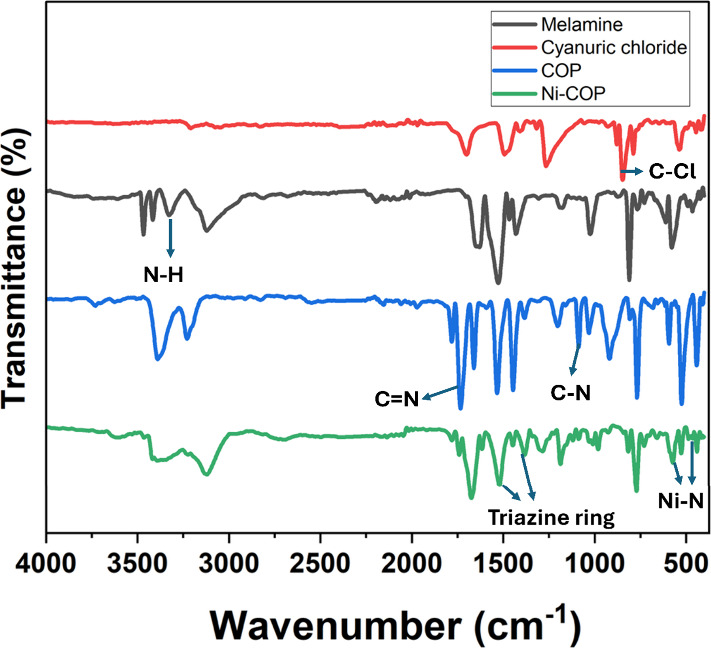
FTIR spectra.

### XRD Studies

XRD analysis of the COP synthesized from melamine and cyanuric chloride showed diffraction peaks centered around 2θ values of 10.5°, 18–22°, and 27.7°, which correspond well with the characteristics of a melamine-cyanurate hydrogen-bonded framework (Figure 2). According to JCPDS 05–0127, the peak at 10.5° corresponds to the (100) plane of the triazine network and the (002) plane at 27.7° corresponds to the π–π stacking between the adjacent aromatic layers.^13,14^ The broad peaks with moderate intensity indicate the partial crystallinity of the COP. The crystallite sizes of COP and Ni-Cop calculated using the Scherrer equation were approximately 44 and 37 nm, respectively.

**Figure 2:**
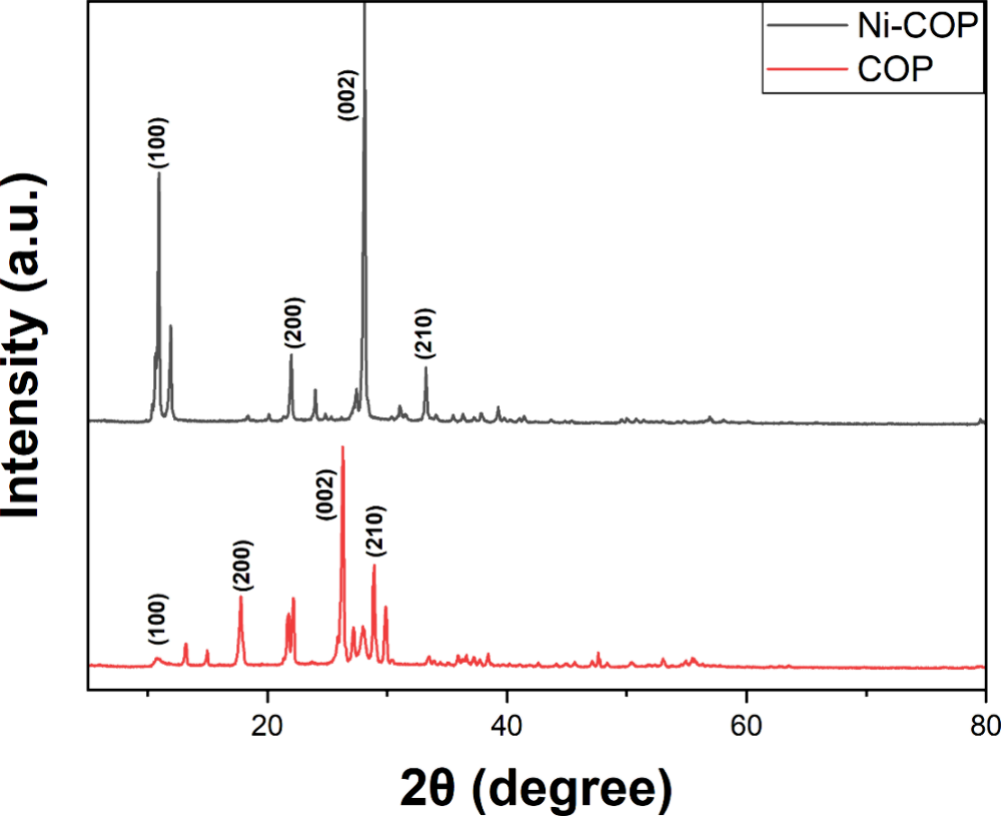
XRD patterns of COP and Ni-COP.

After nickel incorporation, the Ni-COP sample showed noticeable changes in the diffraction pattern, which include the appearance of a sharp peak around the 30–35° region. These features suggest the modification of the structural order of the polymer framework due to the interaction between nickel and nitrogen-rich sites. Additionally, the disappearance of some peaks can be attributed to the structural rearrangement and coordination between the metal and the nitrogen centers. However, the presence of the main characteristic peak around 27.7° corresponding to the (002) plane indicated that the key structural features of the triazine framework were preserved even after nickel incorporation.

### Morphological Studies

SEM analysis showed significant morphological differences between the COP and nickel-doped COP. SEM images of the COP showed a porous, irregular morphology characteristic, which is desirable for ion mobility in supercapacitor applications (Figure 3a). SEM images of the Ni-COP, upon Ni incorporation, showed a denser, more granular morphology, signifying the successful incorporation of nickel species (Figure 3b). This modification should improve electrochemical performance by combining the high surface area of the COP and nickel’s redox activity, thus making Ni-COP a promising electrode material for supercapacitors. The EDS analysis of Ni-COP confirms the successful incorporation of Ni into the COP matrix. The material shows 27.43 wt % nitrogen, which can enhance electrical conductivity (Figure S2).

**Figure 3:**
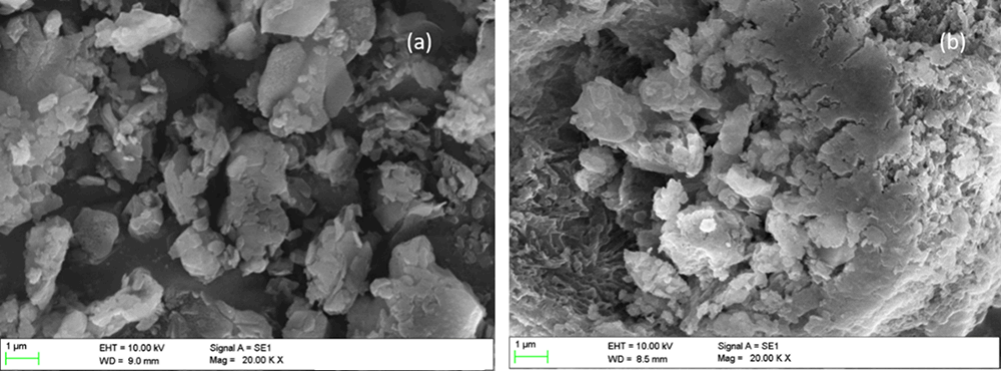
SEM images of the (a) COP and (b) Ni-COP.

### BET Analysis

The nitrogen adsorption–desorption isotherms recorded at 77 K helped in analyzing the porous nature of both the pristine material and Ni-COP. The COP sample exhibited a higher adsorption capacity compared to that of Ni-COP, which was an indication of a higher pore volume.^17^ The pore volumes were found to be approximately 0.006 cm^3^ g^–1^ for COP (Figure 4a) and 0.0025 cm^3^ g^–1^ for Ni-COP (Figure 4b). The pore size distribution analysis showed that mesopores dominated the COP structure with an average pore diameter of about 25 nm, whereas Ni-COP exhibited a broader distribution with a larger pore diameter of ∼ 60 nm. The increase in the pore diameter after nickel incorporation can be attributed to the structural rearrangement of the polymer framework and the smaller pores being occupied by nickel species. The resulting mesoporous structure can help enhance the ion diffusion, which is crucial for the overall performance of the supercapacitor electrode material.

**Figure 4:**
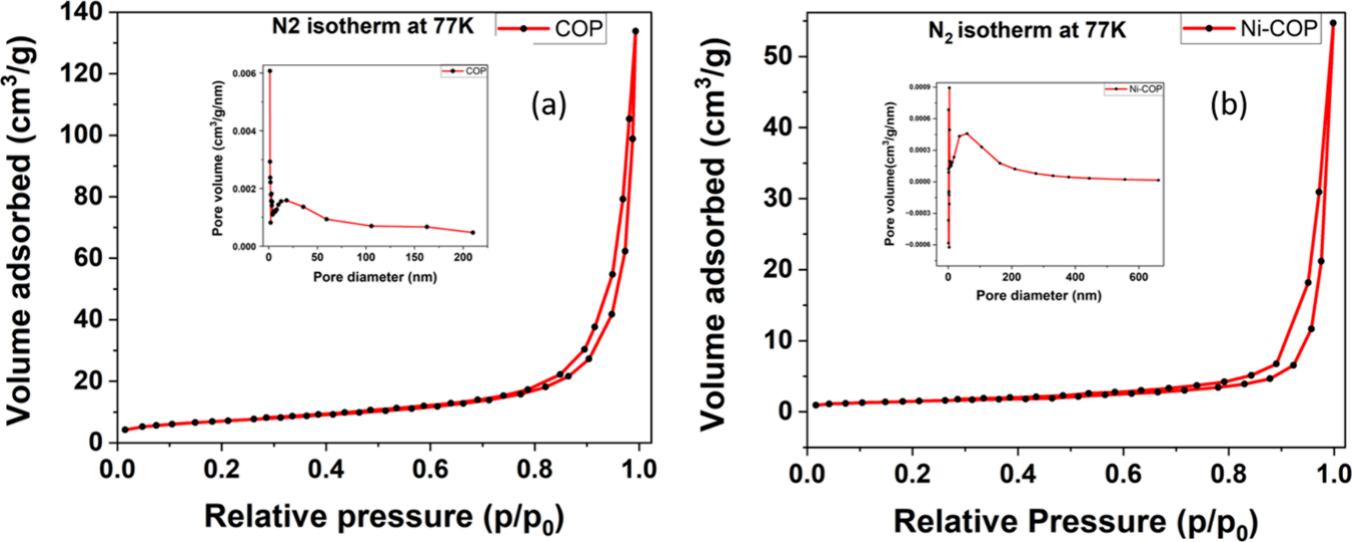
BET results of (a) COP and (b) Ni-COP.

### XPS Studies

XPS was employed to investigate the elemental composition and chemical states of the Ni-COP sample. The survey spectra (Figure 5a) confirmed the presence of all the required elements like C, N, O, and Ni, indicating the successful incorporation of nickel into the COP framework. The high-resolution C 1s spectrum (Figure 5b) was deconvoluted into multiple peaks representing different chemical states of carbon. The peak present at 284.6 eV corresponds to C–C/C=C bonds, arising from the aromatic carbon framework, which is the backbone of the COP structure. Another peak around 286.0 eV was indexed to C–N bonds, indicating the presence of nitrogen-containing functional groups within the framework. The peak at a higher binding energy of about 288.5 eV was attributed to C=N/C=O functional groups, suggesting the existence of heteroatomic groups.^19^

**Figure 5:**
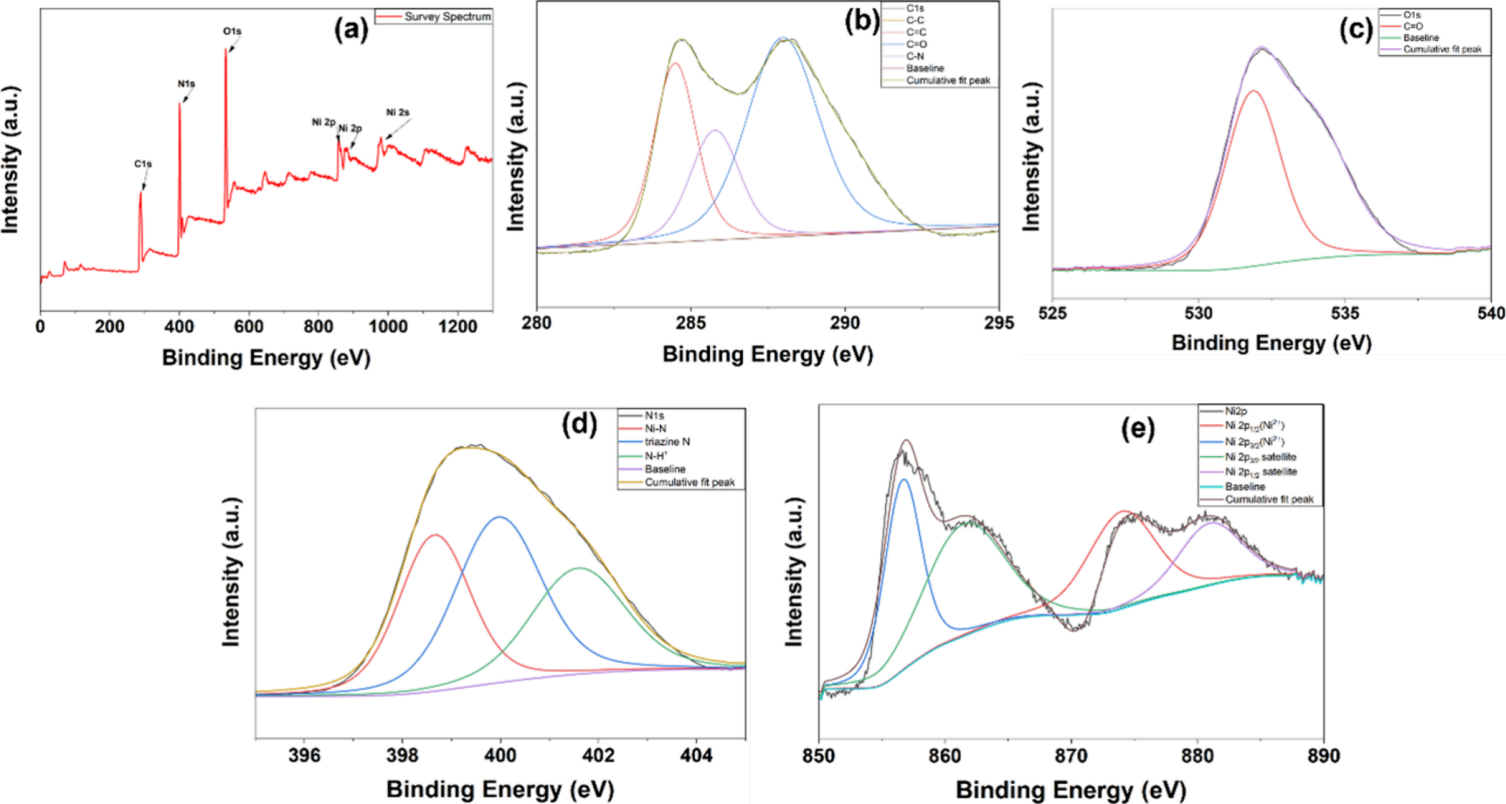
(a) Survey spectrum, (b) C 1s spectra, (c) O 1s spectra, (d) N 1s spectra, and (e) Ni 2p spectra of Ni-COP.

The O 1s spectrum (Figure 5c) exhibited a prominent peak around 531–532 eV, which was attributed to the C=O functional group and may arise from mild oxidation when exposed to the atmosphere.^20^ The N 1s spectrum (Figure 5d) revealed multiple nitrogen chemical states. The peak at 398.5 eV was attributed to the Ni–N coordination, suggesting an interaction between the incorporated nickel and the nitrogen present in the COP framework.^21^ The peak near 399.8 eV was assigned to pyridinic or triazine-type nitrogen, and the peak around 401.5 eV was indexed to protonated or graphitic nitrogen species.^22^

The Ni 2p spectrum (Figure 5e) exhibited two main peaks at approximately 855 eV (Ni 2p_3/2_) and 873 eV (Ni 2p_1/2_), accompanied by their satellite peaks. These are the typical features of Ni^2+^ species, confirming the formation of coordination between nickel and nitrogen. The presence of satellite peaks further confirmed the formation of Ni^2+^ in a coordinated environment.

### Electrochemical Evaluation

All of the electrochemical evaluations were done using 0.1 M H_2_SO_4_ as the aqueous electrolyte. A CV analysis at 5–100 mV/s scan rates detected the electrochemical behavior of pristine COP and Ni-doped COP (Ni-COP) electrodes through the results depicted in Figure 6. During CV tests of the COP electrode, the quasi-rectangular CV curves indicate electric double-layer capacitor behavior at various scan rates (Figure 6a). CV data analysis shows that specific capacitance (C_s_) values decrease as the scan rate increases from 20 F/g at 5 mV/s to 18 F/g (10 mV/s), 15.5 F/g (20 mV/s), 13 F/g (50 mV/s), and 11 F/g (100 mV/s) because ion diffusion and electrode utilization get worse at faster rates. Measuring the Ni-COP electrode shows a distinct enhancement in capacitive output through CV data that confirm pseudocapacitive behavior (Figure 5b). Ni-COP demonstrated values of 40 F/g at 5 mV/s followed by 35 F/g (10 mV/s), 30 F/g (20 mV/s), 22 F/g (50 mV/s), and 18 F/g at 100 mV/s (Figure 6b). The implementation of nickel components resulted in improved charge storage capacity because nickel provides additional sites that activate redox reactions. The Ni-COP device benefits from nickel incorporation by retaining higher amounts of capacitance during swift voltage scans than the pristine COP while showing enhanced electrochemical kinetics that promote rapid ion-transport pathways.

**Figure 6:**
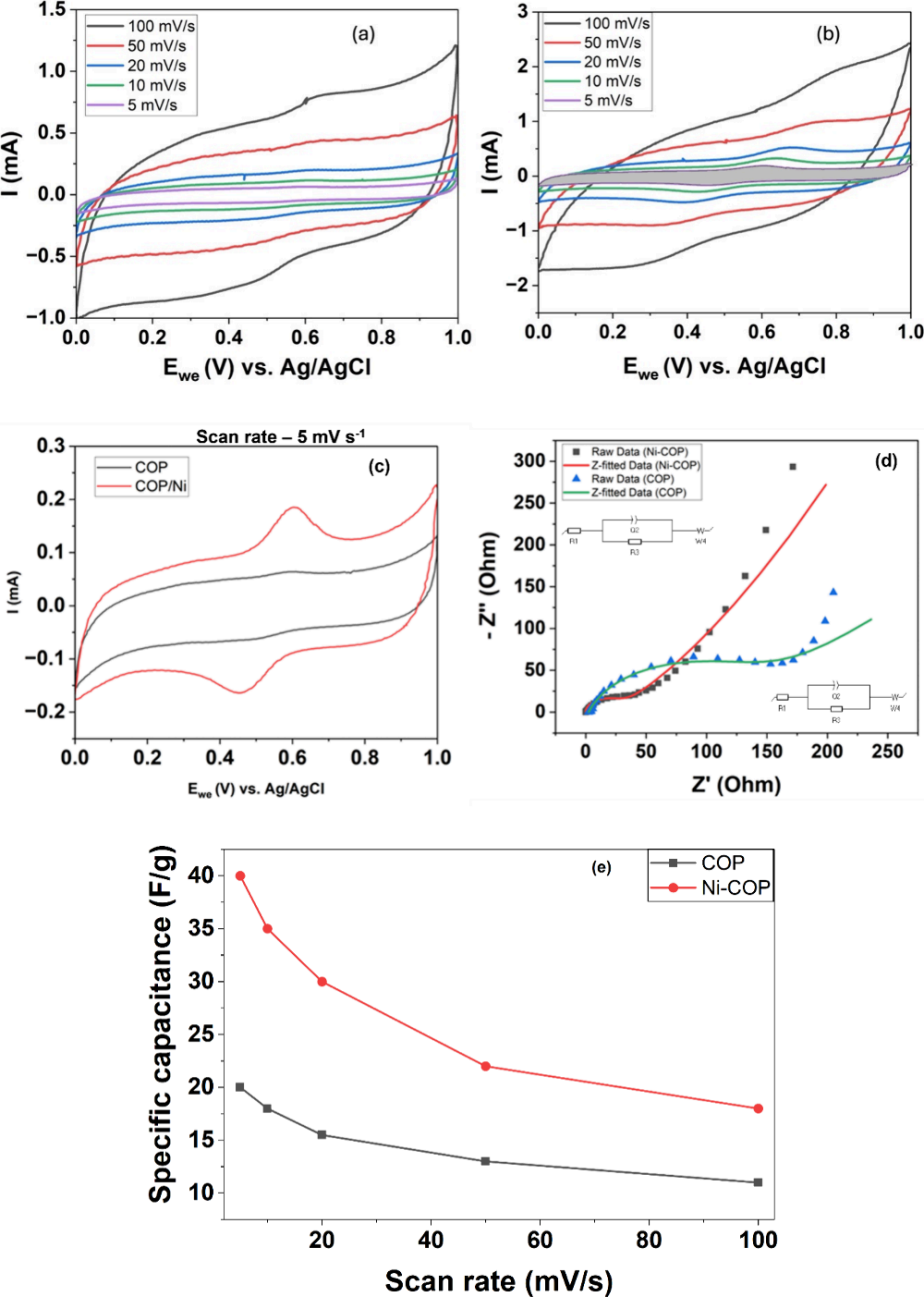
(a) CV of COP in a three-electrode system, (b) CV of Ni-COP, (c) comparison of CV of COP and Ni-COP, (d) comparison of EIS of COP and Ni-COP, and (e) comparison of *C*_s_ vs scan rate.

Electrochemical impedance spectroscopy (EIS) measurements evaluated the charge transfer resistance and ion diffusion conduct maintains for both COP and Ni-COP electrodes as Figure. [Fig fig6]d. Both samples generate Nyquist plots that demonstrate a high-frequency semicircular pattern with subsequent linear behavior at low frequencies, which matches typical capacitive behaviors. The EIS spectra were fitted using the equivalent circuit represented in Figure 6d, wherein R1 (*R*_s_) represents the solution resistance, R3 (*R*_ct_) corresponds to the charge transfer resistance, Q2 represents the constant phase element associated with nonideal double-layer capacitance, and W4 corresponds to the Warburg diffusion element. The semicircle part represents the charge transfer resistance (*R*_ct_) and the linear region showcases Warburg impedance behavior linked to ion diffusion. The Ni-COP electrode produces a minimal high-frequency semicircle with an *R*_ct_ value of 65 Ω, compared to COP, which showed an R_ct_ of 110 Ω, which means its charge transfer resistance at the interface remains very low. Both samples showed a minimal solution resistance (*R*_s_) of about 7 Ω. The Ni-COP plot reveals superior ion diffusion along with improved capacitive performance due to its increased low-frequency slope. The equivalent circuit models derived from the data validate this enhancement as Ni-COP demonstrates a reduced overall impedance value. The electrochemical impedance spectroscopy results demonstrate that nickel addition enhances both COP framework conductivity and electrochemical kinetics to produce Ni-COP, which shows potential for improved high-performance supercapacitor applications.

### Supercapacitor Studies

A symmetric device was fabricated by using the Ni-COP material on both the positive and negative electrodes, and the supercapacitor studies were conducted thoroughly. The Ni-COP electrode’s electrochemical properties were thoroughly assessed through CV measurements as well as GCD techniques and EIS tests using both two-electrode and three-electrode systems, as presented in Figure 7. Under two-electrode conditions (Figure 7a–c), the recorded CV curves displayed a nearly rectangular form along with redox humps, which indicated electric double-layer capacitance and faradaic pseudocapacitive characteristics combined together. CV measurements revealed specific capacitance values of 5 F/g at 100 mV/s, which increased up to 10 F/g when the scan rate fell to 5 mV/s due to improved electrolyte penetration at lower rates (Figure 7a). The GCD profiles of the material demonstrated pseudocapacitive behavior through their quasi-triangular shape as current densities rose (Figure 7c). The specific capacitance values from GCD experiments were 22.85, 19.46, 16.0, 12.45, and 7.54 F/g at 0.1, 0.2, 0.5, 0.8, and 1 A/g, respectively. The electrode delivered the corresponding results for both energy density and power density. The measured energy density values included 3.17, 2.72, 2.22, 1.72, and 1.047 Wh/kg with matching power density results of 13.87, 83.85, 208.11, 417, and 835.5 W/kg. The cycling stability of the Ni-COP-based supercapacitor was thoroughly assessed over 5000 charge and discharge cycles, as shown in Figure 7d. The device showed impressive durability, keeping over 70% of its original specific capacitance at the end of the cycling period. The Coulombic efficiency also remained consistently high, starting near 100% and stabilizing above 80%. This indicates excellent charge and discharge reversibility, along with minimal degradation. These results clearly show that Ni-COP electrodes provide strong, long-term energy storage performance with sustained capacitance and efficiency. This makes them strong candidates for practical supercapacitors in modern energy systems.

**Figure 7:**
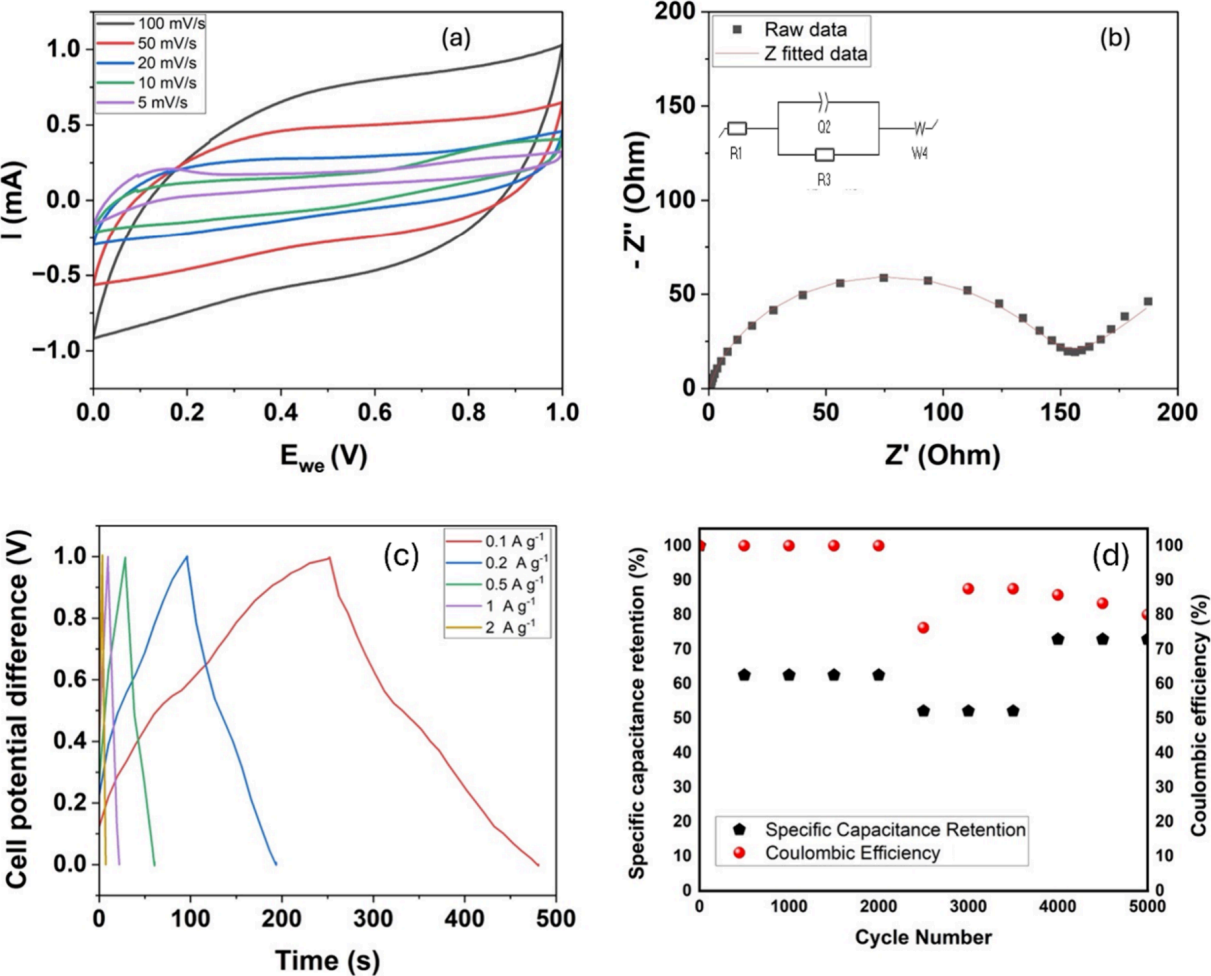
Electrochemical performance of the symmetric device fabricated using Ni-COP electrodes. (a) CV, (b) EIS, (c) GCD, and (d) cycling stability.

### Proposed Mechanism

The proposed mechanism exhibited by the Ni-COP composite is shown in Figure 8. The porous structure of the COP provides easy access to H^+^ ions, and due to the presence of strong bonding in the framework, the structural stability is maintained even at higher cycle numbers. Moreover, the nitrogen-rich triazine groups on the COP provide a secondary interaction with H^+^ ions, thereby enhancing the wettability of the electrode material. The incorporated nickel­(II) coordinates with the nitrogen-rich COPs to produce expanded redox-active sites via interaction between Ni^2+^ and nitrogen functionalities from the polymer. The interaction allows for redox changes between Ni^+^ and Ni^2+^. Furthermore, the Ni ion acts as a bridge between two or more segments of COP, hence creating a connectivity for the movement of ions. Hence, pseudocapacitance behavior due to the combined effect from COP and Ni was observed in the electrochemical studies. More research is needed to establish and understand the organic structure within inorganic materials for energy storage applications.

**Figure 8:**
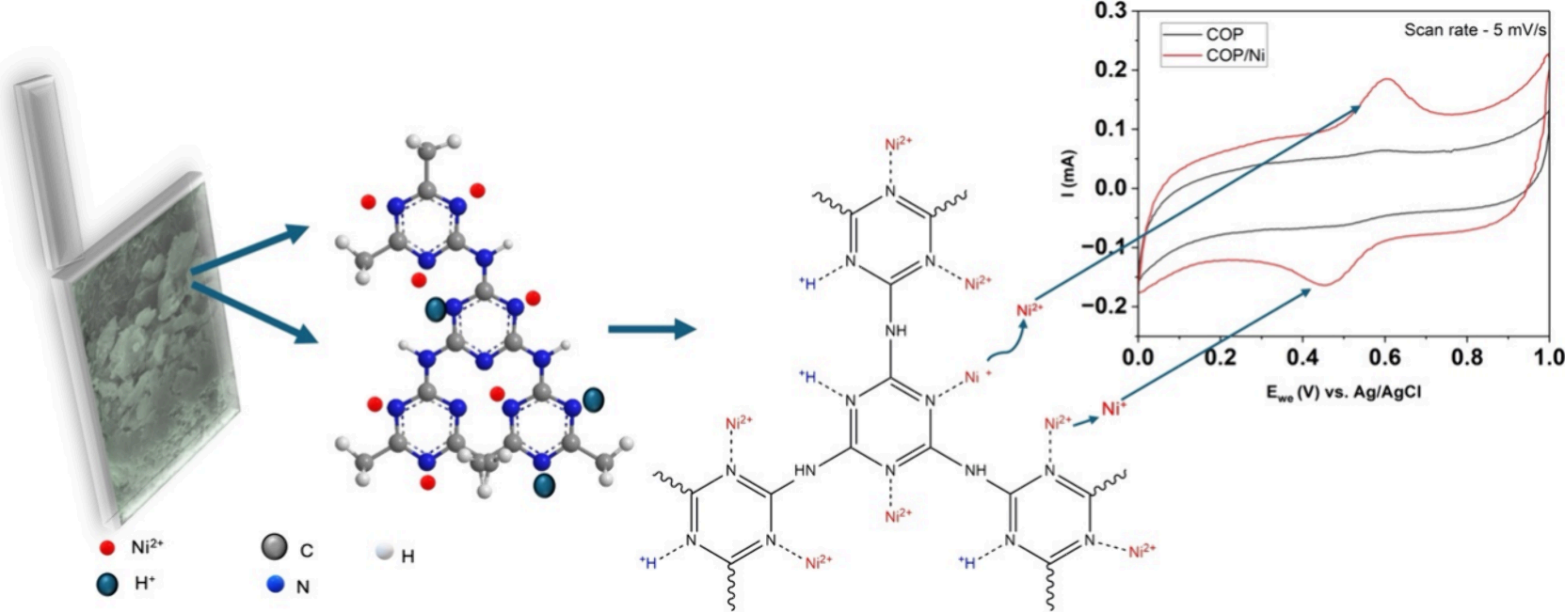
Probable mechanism for the presence of redox peaks in the case of the Ni-COP composite.

## Conclusions

The nickel-doped triazine-based COP prepared in the present work has great potential to serve as a supercapacitor electrode material. It has a high specific capacitance, high power density, and decent cycling stability and retains a specific capacitance of about 70% after repeated charge–discharge cycles. The electrochemical activity is improved by the augmented strength and proper incorporation of the nitrogenous-polymer matrix.

To improve stability and overall capacitance, future research can focus on fine-tuning material incorporation and using different transition-metal ions. The incorporation of these COPs with complementary active materials such as other transition-metal oxides or hybrid composites shows greater promise. It has the potential to diminish the gap between supercapacitors and batteries by increasing both the power and energy densities. Moreover, for these materials to be converted into functional and commercially feasible energy storage devices, efforts have to be put into scaling up the preparation and assembly of devices. The development of high-performance electrode materials for next-generation supercapacitors is aided by the emergence of nickel-doped triazine-based COPs. The development of sustainable high-performance energy storage materials (SDG 13) and the promotion of clean, affordable energy (SDG 7) are two ways that this research contributes to the UN's Sustainable Development Goals.

## Supplementary Material



## Data Availability

The data that support the findings of this study comes under privacy restrictions as it has been used for patent application.

## Notes

EDS mapping of COP and Ni-COP (PDF)
