# Supplementary material for: A Nitrogen-Rich Triazine-Based Covalent Organic Polymer Decorated with Nickel for Supercapacitors
Source: ACS Omega. 2026 Jul 9;11(28):41425–31. doi: 10.1021/acsomega.5c13542 (PMC13393037; doi:10.1021/acsomega.5c13542)
Supplement: Supplementary file 1 [file ao5c13542_si_001.pdf]

A nitrogen-rich triazine-based covalent organic polymer decorated with nickel for supercapacitor

Aditya Bhat[1], Nakul Desai[1], Venkatachalam Hillemane[1]*, Sudhakar Yethadka Narahari[1]*

[1]Manipal Institute of Technology, Manipal Academy of Higher Education, Manipal, 576104, India

*Corresponding authors: venkatachalam.h@manipal.edu, sudhakar.yn@manipal.edu

Supplementary information

EDS mapping of both the COP and Ni-COP revealed a consistent nitrogen rich molecule which indeed shows frameworks essential for supercapacitor applications. The pristine COP exhibits high nitrogen content (51.47% atomic) alongside carbon (31.42%) and oxygen (16.845%), confirming effective incorporation of triazine units that provide abundant active sites for ion coordination and tailored porosity (Fig. S1). Upon nickel incorporation, the Ni-COP shows a slightly reduced nitrogen level (41.05%) with incorporated nickel (4.37%), carbon (26.28%) and increased oxygen (28.31%). This indicates doping and partial nickel oxide formation (Fig. S2). This compositional evolution implies that the nitrogen rich COP matrix offers stable coordination sites enabling uniform nickel incorporation, which introduces redox-active sites critical for enhanced conductivity and pseudocapacitance. The complementary presence of oxygen likely reflects residual functionalities and nickel oxide species, further supporting electrochemical activity. Together, the EDS findings confirm a synergistic material design where optimized nitrogen functionality and stable nickel doping cooperate to improve ion diffusion, electrical conductivity, and overall electrochemical performance, advancing the material's applicability as a high-performance supercapacitor electrode.

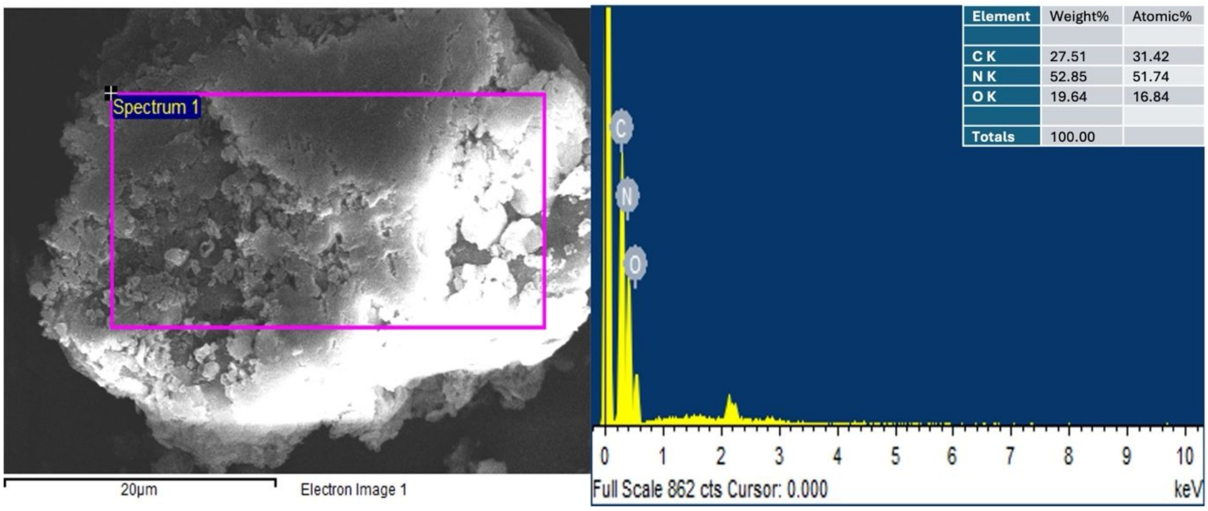

Figure S1. EDS of COP

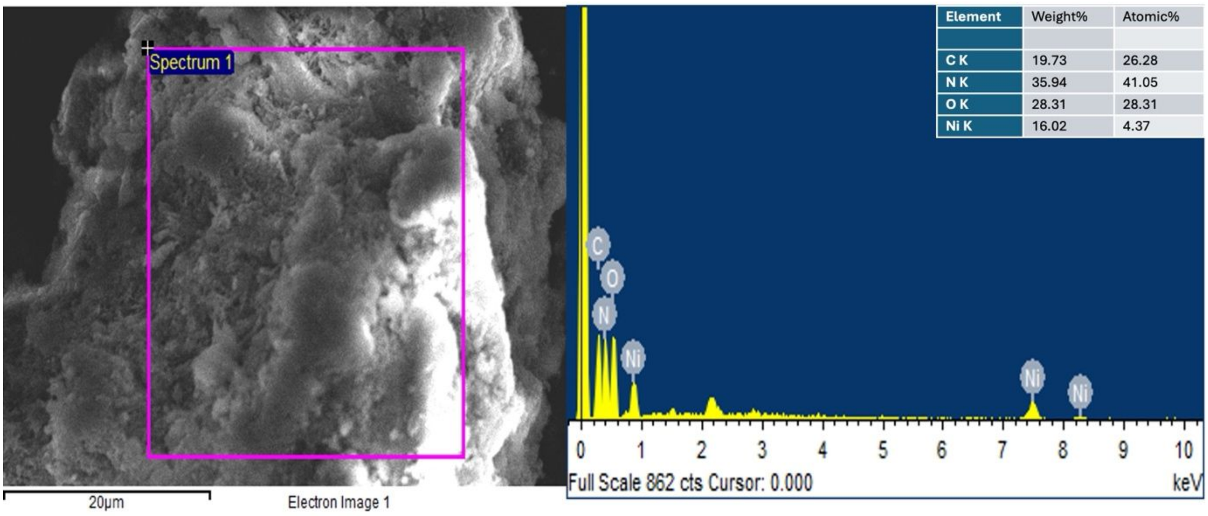

Figure S2. EDS of Ni-COP
